# Molecular Mechanisms of Resistance to Immunotherapy and Antiangiogenic Treatments in Clear Cell Renal Cell Carcinoma

**DOI:** 10.3390/cancers13235981

**Published:** 2021-11-28

**Authors:** Pablo Álvarez Ballesteros, Jesús Chamorro, María San Román-Gil, Javier Pozas, Victoria Gómez Dos Santos, Álvaro Ruiz Granados, Enrique Grande, Teresa Alonso-Gordoa, Javier Molina-Cerrillo

**Affiliations:** 1 Medical Oncology Department, Ramón y Cajal University Hospital, 28034 Madrid, Spain; palvarezb@salud.madrid.org (P.Á.B.); jchamorro@salud.madrid.org (J.C.); mariavictoria.san@salud.madrid.org (M.S.R.-G.); Javier.pozas@salud.madrid.org (J.P.); agranados@salud.madrid.org (Á.R.G.); 2 Urology Department, Ramón y Cajal University Hospital, Alcala University, 28034 Madrid, Spain; vgomezd@salud.madrid.org; 3 MD Anderson Cancer Center, 28033 Madrid, Spain; egrande@oncomadrid.com; 4 Medical Oncology Department, Ramón y Cajal University Hospital, Medical School, Alcala University, 28034 Madrid, Spain

**Keywords:** renal cell cancer, treatment resistance, immunotherapy, angiogenesis, tumor microenvironment

## Abstract

**Simple Summary:**

Renal cell carcinoma is particularly characterized by its high vascularization and dense immune cells infiltration. The angiogenesis blockade in combination with immune checkpoint inhibitors have supposed milestones in the treatment landscape of this tumor. This article gathers the available data on the mechanisms of resistance to current treatments, as well as new strategies under development to overcome these resistances.

**Abstract:**

Clear cell renal cell carcinoma (ccRCC) is the most common histological subtype arising from renal cell carcinomas. This tumor is characterized by a predominant angiogenic and immunogenic microenvironment that interplay with stromal, immune cells, and tumoral cells. Despite the obscure prognosis traditionally related to this entity, strategies including angiogenesis inhibition with tyrosine kinase inhibitors (TKIs), as well as the enhancement of the immune system with the inhibition of immune checkpoint proteins, such as PD-1/PDL-1 and CTLA-4, have revolutionized the treatment landscape. This approach has achieved a substantial improvement in life expectancy and quality of life from patients with advanced ccRCC. Unfortunately, not all patients benefit from this success as most patients will finally progress to these therapies and, even worse, approximately 5 to 30% of patients will primarily progress. In the last few years, preclinical and clinical research have been conducted to decode the biological basis underlying the resistance mechanisms regarding angiogenic and immune-based therapy. In this review, we summarize the insights of these molecular alterations to understand the resistance pathways related to the treatment with TKI and immune checkpoint inhibitors (ICIs). Moreover, we include additional information on novel approaches that are currently under research to overcome these resistance alterations in preclinical studies and early phase clinical trials.

## 1. Introduction

Renal cell carcinoma (RCC) represents around 3% of all cancers in adults showing an incidence of more than 400,000 cases and being responsible for approximately 175,000 deaths worldwide in 2020 [1,2,3]. Approximately 25% of patients present with metastatic disease at initial diagnosis and between 20–40% relapse after nephrectomy for localized disease [4]. Overall, mortality rates for RCC increased until the early 1990s, with rates generally stabilizing or declining thereafter (actually 2.2 renal cancer related deaths per 100.000 population) [5].

Clear cell renal cell carcinoma (ccRCC) is the most common histologic subtype that arises in approximately 75% of RCC [6].

From a molecular point of view, genetic alterations are common in RCC and various genes are involved in its development and progression. Inactivation of the *VHL* gene function by deletion of chromosome 3p, mutation, and/or promoter methylation is a predominant feature of ccRCC [7,8] and leads to abnormal accumulation of hypoxia-inducible factors (HIF-1α and HIF-2α) and activation of the angiogenesis program with increased levels of VEGF [9,10]. However, *VHL* loss itself is insufficient for tumorigenesis, and additional genomic aberrations, such as mutations in 3p-associated genes *PBRM1*, *SETD2*, and *BAP1*; loss of *CDKN2A* and *CDKN2B* genes via focal or arm-level deletion of the 9p21 locus; and alterations in *KDM5C*, *TP53*, *MTOR*, or *PTEN* have been implicated in disease progression and degree of aggressiveness [7].

Over the last 15 years, treatment for metastatic RCC (mRCC) has focused on targeting the VEGF signaling pathway with tyrosine kinase receptor inhibitors (TKI), such as sunitinib, pazopanib, cabozantinib, axitinib or lenvatinib, or monoclonal antibodies that block VEGF, such as bevacizumab. Although VEGF pathway blockade is effective in many patients, it is associated with the development of acquired resistance mechanisms [11,12].

Furthermore, ccRCC is also distinguished as a highly inflamed tumor, with high levels of tumor infiltrating lymphocytes, and a predominant expression of immune checkpoints, such as PD-L1 and CTLA-4 [13,14]. Under this rationale of hypervascularity linked with an immunologically hot tumor microenvironment, inhibitors of the VEGF pathway and the PD-(L)1 axis as monotherapy or in combination, have contribute a noteworthy improvement in terms of survival and quality of life in patients with advanced RCC [6]. Unfortunately, there is an important group of patients who do not respond or lose achieved responses.

In this review we aim to summarize key molecular alterations in RCC to understand the resistance to TKI and immunotherapy treatments, as well as the basis for the development of new drugs that potentially overcome these resistances.

## 2. Molecular Pathways Associated with Resistance to Treatment with Tyrosine-Kinase Inhibitors 

### 2.1. Hypoxia as a Resistance Inductor

Heterogeneity is a pivotal characteristic of RCC, as different genomic and transcriptomic profiles can be observed between primary renal and metastatic lesions [15]. Furthermore, this intratumoral heterogeneity comprises a fundamental feature that hinder efficacy of TKIs. Hypoxia also participates in that inner heterogeneity since RCC tissues show different blood flow conditions.

**Figure 1:**
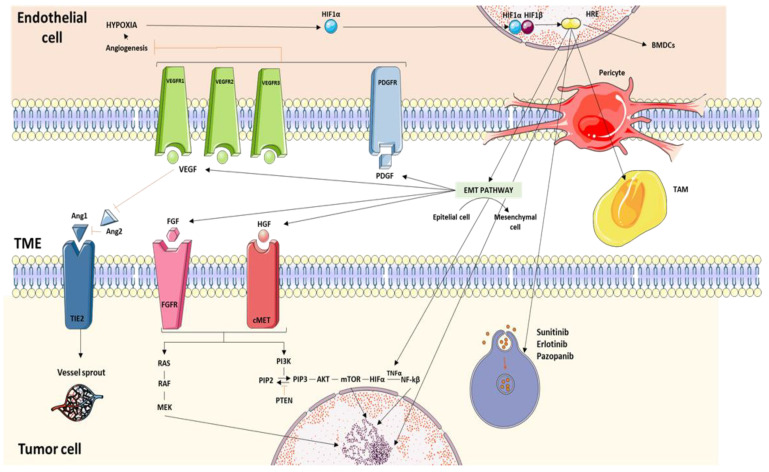
In this figure we illustrate the most preponderant mechanisms of resistance to TKIs: hypoxia-induced activation of alternative proangiogenic pathways, TME factors, EMT, and TKI-induced autophagy.

Anti-VEGF therapies interfere in tumor angiogenesis inducing hypoxic cell death. In consequence, hypoxia enhances epithelial–mesenchymal transition (EMT), causes microenvironmental cells like tumor associated endothelial cells (TECs) and tumor associated macrophages and fibroblasts (TAMs/TAFs) to thrive, increases the expression of proteins involved in lysosomal sequestration of TKIs, interferes with drug penetration, activates many VEGF- and PDGF-independent proangiogenic cascades and alternative pathways that lead to HIF pathway stimulation, and induces alternative modes of vascularization. Moreover, cell glycolysis promoted by hypoxia increases lactic acid levels which is an obstacle for immune cells functions [16]. In this sense, belzutifan, a HIF-2α inhibitor, is currently under development with promising results in disease control rate and duration of response as monotherapy or in combination with other TKI in patients with previously treated mccRCC (NCT03634540, NCT04195750, and NCT 03634540). Indeed, this drug has been approved by the FDA this year, for adult patients with von Hippel–Lindau (VHL) disease who require systemic therapy. Its role in combination with other ICI and in the first line setting is also under research (NCT04736706). However, other novel drugs targeting metabolism, such as telaglenastat, have not shown an additional benefit when analyzed in clinical trails, such as the CANTATA and ENTRATA trials (Figure 1).

### 2.2. Angiogenic Switch

There is robust evidence that describes several non-angiogenic mechanisms which enable tumors to keep growing when angiogenesis is blocked. The first one is known as vessel co-option and lies in the ability of tumor cells to harness normal tissues vessels to maintain oxygen availability [17,18]. It is hypothesized that the initiation of the neoplasm is driven by this angiogenesis-independent strategy, forming the center of the neoplasm. Therefore, co-opted vessels trigger self-apoptosis in order to induce tumor necrosis. Meanwhile, the neoplasm is able to counteract this host defense mechanism by developing neoangiogenesis in the periphery. This process also allows the tumor to initiate metastatic invasion [17].

Vasculogenic mimicry is another less common alternative mode of vascularization that consists in forming channels to provide oxygen to tumor cells. These channels are formed by the tumor cells itself, which can simulate endothelial cells by increasing matrix metalloproteinases in order to modulate tumor microenvironment. This process was mainly described in aggressive melanomas [19].

Another noteworthy way of vascularization is intussusceptive angiogenesis, where no endothelial proliferation is needed and therefore is difficult to counteract with anti-angiogenic drugs. This mechanism is complex and poorly understood since it happens within preexisting vessels. It starts with the interaction of the vessels of opposite walls, forming an interendothelial junction at their edge in the “kissing contact” process. Mesenchymal stem cells, pericytes, and myofibroblasts come into play, taking up the gap formed by the new vessels, creating a new extracellular matrix, and forming the interstitial pillar. Hence, two new transvascular pillars are formed without endothelial proliferation. This mode of vascularization is a rapid and efficient procedure to expand existing vasculature [20,21].

### 2.3. Epithelial–Mesenchymal Transition (EMT)

Epithelial–mesenchymal transition is a well-studied process where the tumor is skilled to change the phenotype of polarized epithelial cells to a mesenchymal one through different molecular and biochemical changes. These empowered cells unhitch from the primary site and invade peritumoral tissues as well as systemic circulation in order to spread across distant places. In addition to these migratory functions, EMT also awards higher resistance to apoptosis and increases extracellular matrix [22].

Sunitinib has different ways to enhance EMT. One of the main pathways that unleash and orchestrate EMT is HIF1-α, accompanied by other molecular pathways such as HGF, EGF or PDGF. HIF-α increases the expression of ZEB1 and ZEB2 which facilitates loss of adhesion of epithelial cells by repressing E-cadherin. [23,24]. Snail and Slug are proteins that participate as well in E-cadherin repression. Sunitinib can favor invasiveness and progression of renal cell carcinoma by stimulating Snail expression and subsequent E-cadherin inhibition. The Akt/GSK3/β–catenin pathway also promotes EMT when activated by cytokines like IL-6, IL-8, and TNF-α [25].

EMT also participates in sarcomatoid differentiation in RCC patients by N-cadherin, Snail and Sparc stimulation and dissociation of β- catetin from cell membrane [26].

### 2.4. Activating Bypass Pathways

#### 2.4.1. VEGF

Sustained treatment with antiangiogenic therapeutics would conduct enhancement of alternative cell signaling pathways that avoids TKIs’ effect. Between the VEGF receptors, VEGFR2 has been the main target for primary TKIs designed, leaving free activity to other VEGFR proteins like VEGFR 1 and VEGFR 3. Furthermore, there are some non-VEGF alternative pathways that allow the tumor to uphold its growth [27].

#### 2.4.2. PTEN

Phosphate and tensin homolog (*PTEN*) are tumor suppressors that have a down regulating function over PI3K/Akt/mTOR pathway. Even though *PTEN* mutations are rarely described in RCC [28], studies have demonstrated that patients with resistance to sunitinib show low expression of *PTEN*, thus constitutively Akt/mTOR expression.

#### 2.4.3. FGF

FGFR pro-angiogenic function is led by upregulation of MAPK/ERK, PI3K/Akt and STAT pathways as well as IP3 and DAG and PKC signaling. Upregulation of FGF2 has been directly related to resistance to sunitinib and constitutes one of the major growth factors able to drive sunitinib resistance. Sunitinib is able to suppress phosphorylation of MEK1/2 and ERK 1/2 conducted by VEGF. However, when FGF2 is overexpressed, strong phosphorylation of MEK ½ and ERK1/2 occurs despite sunitinib administration [29].

#### 2.4.4. Axl and c-MET

Both Axl and c-MET are implicated in antiangiogenic resistance of VEGF targeted therapies and are also related to poor prognosis and decreased overall survival [30,31,32]. Zhou et al. studied the relation between sunitinib resistance and Axl and MET pathways. They demonstrated that in the first phases of treatment, it is able to suppress MET function, but when sunitinib is administered chronically, MET activity is enhanced. Moreover, this activity is maintained once sunitinib is withdrawn. They also proved that treatment with sunitinib increased Axl protein levels. Both Axl and MET are able to promote angiogenesis through activation of ERK and PI3K/AKT signaling and increment of VEGF secretion. Furthermore, sunitinib stimulates Axl and MET dependent EMT and favors cell migration and invasion [30].

#### 2.4.5. TNF-α

Tumor necrosis factor (TNF- α) pathway is involved in multiple physiologic functions like immune response or hematopoiesis, but also plays a key role in tumor pathogenesis. For instance, it is implicated in EMT, activating the nuclear factor κB (NF-κB) pathway through the binding of TNF receptor 1 (TNFR1) and GSK3β activation [33,34].

The involvement of TNF- α in acquired resistances of certain treatments had already been hinted at in breast and lung cancer [35,36], but its implication in RCC remained scarcely explored. In 2020, Hwang et al. discovered that tumor tissues that have acquired TKI resistance express high expression of *TNFR1SF1A* gene. They also related high-*TNFR1* expression in intrinsic-resistance tumors as well as sarcomatoid dedifferentiation [37]. Nevertheless, to which extension TNF-α is involved in TKI resistance in RCC remains to be elucidated and further studies are needed.

#### 2.4.6. Angiopoietin/Tie Pathway

Ang/Tie is a key signaling cascade which constitutes a significant alternative antiangiogenic pathway able to regulate endothelial maturation and vascularization. Angiopoietin 2 (Ang2) has a dual function depending on VEGF presence. When VEGF is inhibited, it binds to Tie2 and inhibits Ang1/Tie2 pathway, consequently promoting vascular degradation and cell death. Wang et al. demonstrated that at the beginning of treatment with sunitinib, the levels of Ang 2 decreased progressively, as long as the tumor was sensitive to sunitinib. Inversely, they showed that patients with sunitinib resistance expressed elevated Ang 2 levels. This fact was correlated with tumor progression, acting Ang2 as an angiogenic escape mechanism [38,39].

#### 2.4.7. Enhancer of Zeste Homologue 2 (EZH2)

The enhancer of zeste homologue 2 (EZH2) is a histone methyltransferase that participates in the methylation of lysine 27 on histone 3 producing gene repression [40].

EZH2 is one of the major epigenetic mechanisms of resistance to TKI in RCC. It enhances EMT, impeding the expression of E-cadherin and therefore favoring invasiveness and migration [40]. Adelaiye et al. exposed in their studies that EZH2 overexpression leads to methylation of promoter regions of anti-angiogenic factors and subsequently favors tumor vascularization and therefore sunitinib resistance. Furthermore, EZH2 can induce adaptive kinase reprogramming through epigenetic changes, allowing tumor cells to find alternative pathways such as FAK, SCR, MET, FGFR2, EGFR, IGF-1R, and ERBB2 [41]. Nevertheless, this resistance mechanism can be counteracted by dose escalation [42].

### 2.5. Lysosomal Sequestration of TKIs

Lysosomal sequestration is the process by which sunitinib is accumulated within the lysosome structure. Most TKIs can traverse lysosomal membrane easily because they are weak bases. Once the molecule is internalized, it finds an acid environment achieved by proton pumping vacuolar ATPases. This environment protonates the molecule and sequestrates it inside the lysosome. Therefore, it is unable to exert its function [43].

Certain TKIs, such as erlotinib and pazopanib, can also be exposed to lysosomal sequestration [44]. Sorafenib comprises a different kind of molecule with differential characteristics that does not permit free travel across lysosomal membranes. Because of this fact, other lysosomal sequestration mechanisms have been proposed for Sorafenib. It was demonstrated that drug pumps like ABC transporter P-glycoprotein can mediate not only sunitinib sequestration but sorafenib too. In the frame of this thinking, P-gp inhibitors like verapamil or elacridar have been studied in preclinical models of CCR showing enhancement of antitumor activity of sunitinib [45,46,47].

Moreover, lysosome sequestration is a multidrug resistance (MDR) mechanism that can lead to a feedback process where the exposure to tyrosine kinase inhibitors reinforces lysosome biogenesis. The increased lysosomal gene expression and lysosomal enzyme activity lead to augmented drug sequestration and MDR. Lysosomal biogenesis seems to be driven by the nuclear transcription of transcription factor EF (TFEB) [48]. This process is ultimately commanded by mTORC1 [49]

### 2.6. Noncoding RNAs (ncRNA) and Single Nucleotide Polymorphisms

Circulating noncoding RNAs have raised interest in many oncologic fields. They have been studied as potential biomarkers in early stages of RCC as well as prognostic and predictive treatment response biomarkers [50,51,52].

Micro RNA (miRNA), a particular class of ncRNA, have been studied as molecules able to carry out TKIs resistance, concretely miRNA-15b, which overexpression has been described as a mechanism of resistance to sunitinib [53]. Other miRNA like miRNA-575, miRNA-642b-3p and miRNA-4430 were detected in cultures of RCC cells resistant to sunitinib [54]. Regulation of miR-141 and miR-429 also contributes to EMT and its development [55].

Le Qu et al. described a sunitinib resistance mechanism based on intercellular transfer by exosomes of long noncoding RNA (lncRNA) called IncARSR. Long-noncoding RNA are a class of ncRNA with a minimum length of 200 bases involved in gene transcription by multiple regulation functions such as recruitment of chromatin-modifying complexes and post-transcriptional modulation [56,57].

Le Qu’s analysis confirmed high levels of lncARSR in sunitinib-resistant RCC tumor cells as well as endothelial cells. LncASRS seemed to be upregulated by the activation of the AKT pathway and ultimately the inhibition of FOXO1 and FOXO3a. LncASRS is packed into exosomes via heterogeneous nuclear ribo-nuclear protein A2B1 (hnRNP A2B1) and afterwards transferred to surrounding cells disseminating sunitinib resistance. The authors hypothesized and confirmed that lncASRS functioned like competing endogenous RNA (ceRNA) for miR-34 and miR-449, whose targets are Axl and c-MET. This competitive binding increased the expression of Axl and c-MET, hence the stimulation of STAT3, AKT, and ERC pathways and subsequent sunitinib resistance.

Single nucleotide polymorphisms (SNPs) are the most common genetic variation and are defined as a single base pair variation that reaches at least 1% of the population. SNPs related to sunitinib pharmacokinetics (ABCB1, NR1/2, and NR 1/3) and pharmacodynamics (VEGFR3 and FGFR3) had already been described by Beuselinck et al. as determinants of sunitinib outcome in RCC patients [58]. Their effect in CYP3A4 is essential in the metabolism of sunitinib. SNPs in NR1I2 and NR1I3 suppressed CYP3A4 function and were associated with shorter PFS. Inversely, SNPs in CYP3A4 were associated with increased PFS as a result of increased metabolism of sunitinib [58,59].

### 2.7. Tumor Microenvironment Factors Related to Resistance to TKIs

Tumor microenvironment (TME) is constituted by several components such as the tumor cells itself, extracellular matrix (ECM), fibroblasts, vascular endothelial cells, immune cells, and several other stromal cells. Tumor microenvironment is an essential participant of tumor progression and maintenance of its pathogenesis [60].

Robust evidence has been constructed in recent years supporting the importance of tumor microenvironment in development of resistance to TKIs.

#### 2.7.1. Tumor Endothelial Cells (TECs)

Tumor endothelial cells are an important element of TME and participate actively in tumor development. They blossom in hypoxic conditions and can also drive resistance to targeted therapeutics. A study reflected that sunitinib was able to increase VEGF and vascular cell adhesion molecule-1 (sVCAM) as well as levels of circulating endothelial cell-related proteins like Ang-2. The increase of these proteins and TECs were described in patients with acquired resistance to sunitinib [61]. Notch ligand Delta-like 4 (Dll4) has been also related to TECs and the expression of this pathway exerts downstream inhibition of VEGF [62,63].

#### 2.7.2. Bone Marrow-Derived Proangiogenic Inflammatory Cell Recruitment

Hypoxic conditions lead to recruitment of different bone marrow-derived cells (BMDCs) and it is known that this environment is enhanced by antiangiogenic agents. BMDC can participate in the formation of a premetastatic niche environment by crafting new vessels that supply oxygen tumor requirements.

Myeloid-derived suppressor cells (MDSC) is a class of BMDCs worth highlighting. This major component of TME is able to induce resistance to TKIs by enhancing VEGF-independent angiogenesis. This is carried out by GM-CSF availability in tumor tissue and is a STAT5 dependent mechanism, since it was objectified that START 5ab (null/null) MDSC were not able to induce sunitinib resistance [64,65].

#### 2.7.3. Pericyte Coverage

By their attachment around blood vessels and expression of proangiogenic factors like VEGF, pericytes promote proliferation and maintenance of tumorigenesis. When they are pathologically activated, abnormal micro-vessel networks embedding the tumor cells are formed. It is known that increase of pericyte coverage favors antiangiogenic resistance enhancing survival of endothelial cells and making them less sensitive to VEGF inhibition [66].

#### 2.7.4. Tumor-Associated Fibroblasts (TAFs)

There is strong evidence that tumor-associated fibroblasts (TAFs) are able to interact with multiple signaling pathways in RCC cells and promote angiogenesis, tumor invasion, and TKI resistance through paracrine mechanisms. For instance, it can enhance HIF-1α accumulation in RCC through CXCR4 upregulation favoring resistance to treatments. CXCR4 is a molecular proangiogenic pathway expressed by many components of TME such as TAFs. This process is induced by *VHL* malfunction, which is inherent to RCC pathogenesis [67,68]. TAFs can promote resistance to anti-angiogenic molecules promoting activation alternative pathways such as MAPK/ERK and Akt [69].

They also interact with interstitial fluid pressure inside the tumor and are capable of nullifying the travel of drugs through tumor cells. They also mediate induction of aggressive phenotypes of RCC as a result of increased recruitment of macrophages and remodeling of TME [70,71].

Crawford et al. showed that TAFs stimulate expression of PDGF-C and consequently generate angiogenesis and treatment resistance [72].

#### 2.7.5. Tumor-Associated Macrophages

Tumor-associated macrophages (TAMs) have been lately attributed an important role in tumor induction and progression. Nevertheless, they can have a twofold function being able to enhance tumor growth as well as produce anti-tumor signals [73]. It is known that hypoxia prompts tumor-associated macrophages to favor tumor progression through secretion of different molecules like MMP-9, CSC chemokines, IL-6, TNF-α, and VEGF which not only promotes angiogenesis but also participate in TME regulation. All this angiogenic storm can aid the tumor to find alternative pathways and lessen the effect of anti-angiogenic therapies [74].

## 3. Molecular Pathways Associated with Resistance to Treatment with Immune Checkpoint Inhibitors 

**Figure 2:**
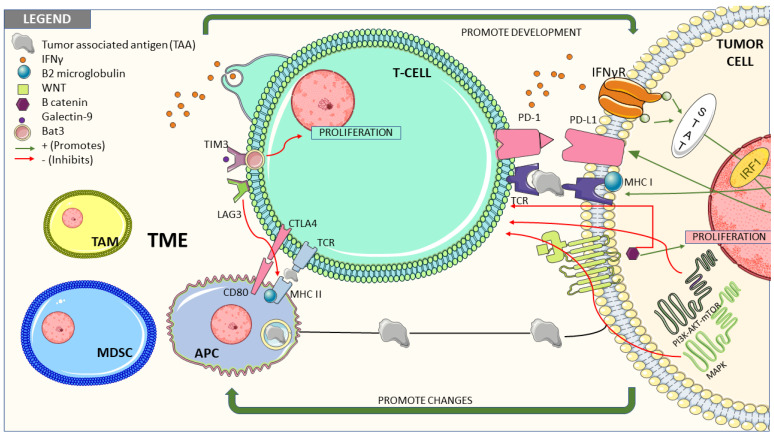
Among the key mechanisms described we can mainly distinguish tumor-intrinsic factors and factors associated to tumor microenvironment (TME). In the first subgroup it is important to outline the alterations of antitumor immune response pathways (e.g., aberrant expression of tumor antigens), variations in the antigen presentation pathways (e.g., β2-microglobulin mutations leading to loss of MHC) or defective signaling pathways (e.g., IFNγ-STAT-IRF1 signaling pathway); What is more, these intrinsic factors promote the formation of an immunosuppressive microenvironment through the mutations of functional genes such as Wnt/β-catenin, MAPK, or PI3K-AKT-mTOR pathways and the modifications of the metabolism of TME (e.g., hypoxic conditions); The second subgroup (factors associated to TME) includes the presence of immunosuppressive cells (e.g., MDSCs or TAM) as well as the activation of coinhibitory receptors (e.g., TIM-3, LAG-3).

Many factors have been described as relevant in the resistance to immunotherapy in different tumors, leading to two main forms of resistance (primary resistance and secondary). Primary resistance makes reference to intrinsic resistance (probably related to the tumor) and secondary to acquired resistance (probably related to microenvironment changes) in patients with initial response to treatments. For simplicity, these factors have been classified into “intrinsic tumor mechanisms” and “microenvironment related” (Figure 2).

### 3.1. Tumor Cells-Intrinsic Factors

#### 3.1.1. Interferon Gamma Signaling Pathway

The intrinsic interferon gamma (INFγ) pathway plays a key role in the T-cell response against a tumor antigen. The activation of the INFγ membrane receptor results in the downstream interaction with the Janus Kinase (JAK) signal transducer, the activator of transcription (STAT) and the interferon regulatory factor 1 (IRF1), leading to PD-L1 expression. Genetic disorders in the INFγ signaling pathway have been revealed as resistance-associated to treatment with ICI [75]. Moreover, INFγ enhances MHC-I antigen presentation. In MHC-deficient tumor cells, treatment with INFγ is necessary to express the antigen processing machinery and has been able to induce tumor-specific T-cell responses [76]. INFγ pathway also promote the recruitment of immune cells and has direct effects over the tumoral cells, leading to anti-proliferative and proapoptotic signals [77]. Recently, loss-of-function truncating mutations in genes *JAK1* and *JAK2* have been associated with lack of response to INFγ, as well as PD-1 inhibitors’ inefficacy [78].

#### 3.1.2. Wnt/β-catenin Pathway

The Wnt/β-catenin pathway is associated with different biological processes, such as stem cell development, embryogenesis, cell differentiation, and immune regulation. In most cancers, Wnt/β-catenin is overexpressed. In several tumoral models not including renal cell carcinoma, this overactivation is correlated to absence of T cell gene expression signatures and T-cell exclusion, leading to “immune-desert” tumors, conditioning resistance to immune checkpoint inhibitors. [79,80,81,82]. Wnt/β-catenin is also involved in the regulation of IDO1 and the PPARgamma receptor, both inducing immunosuppressive effects [83]. A role in tumor stemness and dedifferentiation is also well-described [84].

#### 3.1.3. Mitogen-Activated Protein Kinases (MAPK) Pathway

The MAPK pathway is associated with VEGF, IL-6, IL-8, and IL-10 production and has been related with the inhibition of T cell functions and immune cells recruitment. Furthermore, MAPK pathway mediates in the negative regulation of MHC expression and antigen presentation, as well as a reduced responsiveness to the anti-proliferative effects of IFNγ and TNFα [75,85].

#### 3.1.4. PI3K/AKT/m-TOR Pathway

PI3K/AKT pathway has been identified as one of the most altered pathways in ccRCC, following the molecular characterization performed by The Cancer Genome Atlas Program. The loss of expression of PTEN has been pointed out as another relevant alteration [86]. These alterations have been associated with expression of immunosuppressive cytokines and inhibition of the autophagosome, resulting in a decreased T-cell infiltration at tumor sites, poor T-cell recruitment, and failure of T-cell-mediated cell death. PTEN loss has also been correlated with worst outcomes with anti PD-1 inhibitor therapy [87].

#### 3.1.5. Cell Cycle Checkpoint Pathway

Cyclin dependent kinase 4 and 6 (CDK4/6) and their co-factors D-type cyclins are principal drivers of the cell cycle from G1 to S phase and have been associated with tumoral progression. Several studies have emphasized the impact of CDK 4/6 inhibition enhancing the immune response. Thus, the CDK4/6 inhibitor abemaciclib in combination with immune checkpoint blockade had a substantially greater capacity to induce pronounced responses in mouse breast cancer models than either agent alone [88,89]. A substantial IL-2 expression and increased T-cell tumor infiltration was observed in these models and have been connected to the beneficial effect of CDK4 inhibition on antitumoral immunity [90].

#### 3.1.6. Loss of MHC

The loss of MHC I and II molecules favors the tumoral immune escape by incapacitating the T-cells to recognize the tumoral antigens. Many genetic and epigenetic alterations that involves the antigen processing and presenting machinery have been potentially associated with this event. Truncating mutations in the gene encoding B2-microglobulin has shown a loss of expression of MHC I in the cell surface, resulting in an absence of response to ICI in melanoma patients [88,91]. In addition, loss of heterozygosity at the B2-microglobulin locus was associated with lower overall survival in melanoma patients receiving immune checkpoint inhibitors [92].

### 3.2. Tumor Microenvironment Related Factors and their Role in Resistance to Immune Response

#### 3.2.1. T Cells

RCC is one of the most T cell-enriched tumors. The high densities of CD8+ tumor-infiltrating lymphocytes (TILs) is associated with a poorer prognosis, compared to other tumor types [93,94]. Amongst the many hypotheses that underlie this contra-intuitive prognosis on the impact of CD8 in ccRCC, it has been demonstrated that co-expression of PD-1 and LAG-3 induced by a lack of antigen presentation by dysfunctional dendritic cells results in CD8 TILs exhaustion in ccRCC [95].

However, recently the controversial role of tumor infiltrating T cells has started to be clarified. In the phase III trial JAVELIN RENAL 101 (comparing the combination of anti PD-L1 antibody avelumab + TKI axitinib vs sunitinib in monotherapy), an association between large CD8 infiltration and poor PFS in patients treated with sunitinib was observed. However, these outcomes were not reflected in patients treated with the combination, suggesting that CD8 infiltration has prognostic value in TKI-treated ccRCC but loses it when the patient is treated with ICI [96].

In 2017, Giraldo et al. [97] proposed a classification of primary ccRCCs depending on their dominant immune profile. They studied 40 tumors, dividing them in three different profiles: 1. The immune regulated, represented by polyclonal cytotoxic CD8+ PD-1+ Tim-3+ Lag 3+ TILs and CD4+ ICOS+ cells with a Treg phenotype, characterized by highly infiltrated tumors with notable proportion of dysfunctional dendritic cells expressing PD-L1. 2. The immune activated, distinguished by oligoclonal/ CD8+ PD-1+ Tim-3+ TILs, that represented 22% of the patients. 3. The immune silent, enriched in TILs revealing a RIL-like (renal infiltrating lymphocytes) phenotype, constituting the majority of tumors of the cohort (56% of the patients analyze).

The immune regulated and immune activated tumors have been connected with distinctive phenotypic signatures, which confer aggressive histologic properties and high risk of relapse or progression. These findings support the hypothesis that these selected patients could benefit from adjuvant treatment with ICIs [97].

Subsequently, molecular biomarkers evaluated in the IMmotion 150 phase II trial (comparing first line treatment in mccRCC with the combination of atezolizumab + bevacizumab versus standard therapy with sunitinib) showed distinct biological subgroups based on levels of angiogenesis, immune infiltration, and myeloid inflammation. In addition, the subgroup with high expression of the Angio gene signature (Angio^High^) was characterized by higher vascular density and was associated with improved response within the sunitinib arm. The Angio^Low^ subgroup showed better response to atezolizumab + bevacizumab versus sunitinib. Moreover, high expression of the T-effector (T_eff_) gene signature was positively associated with expression of PD-L1 and CD8 T-cell infiltration. The T_eff_^High^ subgroup had an improved ORR and PFS with atezolizumab + bevacizumab compared with T_eff_^Low^ subgroup. High Teff gene signature was also related to improve PFS with atezolizumab + bevacizumab versus sunitinib, and showed no difference with atezolizumab in monotherapy, which can highlight the role of Teff gene signature in response and resistance to immunotherapy. Complementary, differential expression of genes associated with myeloid inflammation within the T_eff_^High^ and T_eff_^Low^ subgroups was observed. Atezolizumab monotherapy had worse activity in the T_eff_^High^Myeloid^High^ tumors compared with the T_eff_^High^Myeloid^Low^ group [98].

Motzer et al. characterized seven molecular subtypes of ccRCC using a large RNA-seq dataset from the IMmotion 151 phase III trial [99]. They identified and refined transcriptionally defined subgroups using non-negative matrix factorization, an unsupervised clustering algorithm. Patient tumors in clusters 1 (Angiogenic/Stromal) and 2 (Angiogenic) were characterized as highly angiogenic, with enrichment of VEGF pathway-related genes. These tumors showed the longest PFS in both treatment arms, suggesting better outcomes regardless of treatment. However, no differences between the combination treatment with atezolizumab + bevacizumab versus sunitinib were observed, which suggests that these groups essentially benefit from treatment with antiangiogenics. Clusters 4 (T-effector/Proliferative), 5 (Proliferative), and 6 (Stromal/Proliferative) were characterized by enrichment of cell cycle transcriptional programs, and lower expression of angiogenesis-related genes. Atezolizumab + bevacizumab treatment showed improved ORR and PFS over sunitinib in tumors from clusters 4 and 5, confirming the contribution of pre-existing intratumoral adaptive immune presence described in these patients. However, cluster 6 was associated with a poor outcome.

At last, cluster 3 (Complement/Ω-oxidation cluster) presents lower expression of both angiogenesis and immune genes and has been associated with poor prognosis. Cluster 7 (snoRNA) is characterized by expression of snoRNA (small nucleolar RNA, a group of RNA molecules of variable length, that guide modifications processes of other RNAs, mainly ribosomal RNA maturation), especially C/D box snoRNAs which have been implicated in alterations of epigenetic and translation programs. This last cluster improved PFS with atezolizumab + bevacizumab, but the biological basis of this effect remains to be elucidated.

Additionally, IDO-1 upregulation was described as a key driver of T cell nutrient deprivation. IDO-1 overexpression in tumor endothelial cells is associated with better response and PFS in patients treated with nivolumab and has been proposed as a new biomarker [100].

#### 3.2.2. Innate Immune System

Macrophages can undergo M1 (classical) or M2 (alternative) activation in result of the inflammatory triggering signal. The M1 type are characterized by producing high levels of inflammatory cytokines, such as IL-12, IL-23, and IL-6. M2 macrophages can be subdivided into different subsets called M2a, M2b, M2c, and M2d [101,102]. Th2 cytokines IL-4 and IL-13 stimulate the macrophages to develop M2a phenotype; M2b are induced by activation of Toll-like receptors; and IL-10 polarizes the M2c subtype. M2d subtype is also known as tumor-associated, due to the ability of tumor cells to switch the potential phenotype of macrophages into this subtype. Tumor associated macrophages express multiple receptors or ligands of immune inhibitory pathways, such as PD-L1, PD-L2, and B7-1 [101]. In RCC, poor survival outcomes have been identified in tumors with high expression of anti-inflammatory macrophage phenotype (M2) [103]. Moreover, extensive tumor-associated macrophage (M2d) infiltration into the RCC microenvironment leads the recruitment of Tregs to the tumor site by secreting CCL20 or CCL22 and has been linked with enhancement of angiogenesis, tumor proliferation, and metastatic cellular migration and invasion.

#### 3.2.3. B Cells and Tertiary Lymphoid Structures

B cells and tertiary lymphoid structures (TLS) have recently arisen as an important feature in cancer biology. B cells have been analyzed within the tumor and the microenvironment, showing a strong memory response against tumor associated antigens [104]. Bregs are a specific population of B cells with a regulatory role that have been marked as inmunosupressive cells, due to their capacity to secrete inhibitory molecules, like IL-10 and TGFβ, which regulate T-reg differentiation [105]. In ccRCC, higher expression of B cell related genes, measured by microarrays profiling of baseline tumor samples, have been associated with better response to ICIs [106]. In sarcoma, a cluster of patients (known as “immune and TLS high”) which predominantly express the B lineage signature, has demonstrated a significant improvement in life expectancy with anti PD-1 treatment [107].

Tertiary lymphoid structures are ectopic lymph-like structures whose structure varies from an aggregation on B and T cells to more complex structures. Generally, these TLS are constituted by a T cell zone with mature dendritic cells covering a follicular zone rich in proliferating and differentiating B cells. These structures play an important (and still largely unknown) role against tumor immunity and are associated with better prognosis in patients with several cancers, including ccRCC. Typically, these structures can develop a niche which supports the appearance of transformed cells and activated T regs, favoring the immune response [93,108].

#### 3.2.4. Proinflammatory Cytokines

The RCC microenvironment is associated with pro-inflammatory conditions. Among the factors associated with this fact, the release of pro-inflammatory molecules and cytokines induced by tissue damage emerges as the most important one. Upper concentrations of molecules, such as adenosine triphosphate, IL-6, IL-8, macrophage inflammatory protein 1-alpha, tumor necrosis factor alpha (TNFα), or IFNγ promote the angiogenesis, genomic instability, cellular proliferation, and the epithelial–mesenchymal transition, as well as increase the recruitment of immune cells, leading to a pro-tumorigenic microenvironment.

Furthermore, it is important to notice that this recruitment promotes immunosuppression leading by the increased expression of PD-1 on T cells which is induced by IFNγ also [109]. This sustained expression of PD-1 is responsible for T cell exhaustion via the SHP2 recruitment. Transcriptional factors such as STAT-3 and IRF1, induced by pro-inflammatory conditions, also modulate the expression of PDL1 and PDL2, favoring this exhaustion process. Additionally, IL-1, IL-6, IL-11, IL-17, and TNF alpha promote Treg expansion and increase T cell exhaustion [110,111].

#### 3.2.5. Hypoxia

RCC is characterized as being one of the most vascularized tumors. However, this vascularization is composed of fragile, disorganized vessels, causing an erratic nutrient and oxygen intake, which leads to hypoxia and a lower pH, facilitating tumor progression [112]. Furthermore, hypoxia induces the activation of different genes, which are involved in differentiation of tumor associated macrophages, Treg recruitment and infiltration of myeloid-derived suppressor cells. These immune structural changes favor the inhibition of T cells [113,114]. Furthermore, HIF-1a and HIF-2a induce increased expression of PD-L1 in tumor cells [115,116]. An immune escape pathway is developed by increased levels of HIF-1 and HIF-2, which enables the generation of VEGF, which in turn increase the expression of the immune checkpoints CTLA-4, TIM-3, and LAG-3 on T cells, and PD-L1 on dendritic cells [114,117]. Finally, hypoxic tissues are enriched in adenosine, which suppress the effect of T cells, contributing to immune escape [118].

#### 3.2.6. Protein Polybromo-1(PBRM-1) Expression

PBRM-1 is a specific subunit of the PBAF form of the SWI/SNF chromatin remodeling complex. Loss-of-function mutations in this complex are recurrent in many cancers, including ccRCC, which appears in around 40% of patients [119,120]. In ccRCC, low expression of PBRM1 and high tumor grade imply a worse prognosis. In vitro studies performing the inactivation of PBRM1 using CRISPR-Cas9, have shown a larger production of chemokines in response to IFNγ, which recruits effector T cells and promotes sensibilization of treatment-resistant mouse melanoma cells to immunotherapy [119]. Other studies involving whole exome sequencing have remarked that the loss-of-function mutation in the *PBRM1* gene has been linked with improved PFS and OS in patients receiving antiPD-1 treatment [121,122,123]. However, recent studies have demonstrated that ccRCC with low expression of PBRM-1 are related with lower CD4-CD8 tumor infiltration, lower expression levels of CXCL10, CCL12, ICAM-1, and other cell migration-related molecules, and in the end, with poorer outcomes with anti-PD1 treatment compared with PBRM-1 high tumors [124]. These new findings reveal the potential of PBRM-1 as a therapeutic target.

#### 3.2.7. Immune Escape Related to Other Immune Checkpoints

T-cell immunoglobulin and mucin domain 3 (TIM-3) is a type I trans-membrane protein that was originally discovered in an effort to identify novel cell surface molecules that would mark IFN-γ-producing Th1 and Tc1 cells. Tim-3 plays a key role in inhibiting Th1 responses and the expression of cytokines such as TNF and INF-γ, leading to the suppression of tumoral immune response [125]. On T-cell activation, TIM-3 is recruited to the immunological synapse with B-associated transcript 3 (Bat3) bound to the cytoplasmic tail of TIM-3. When TIM-3 is engaged by a ligand, in most cases galectin-9, the conserved tyrosine residues in the cytoplasmic tail become phosphorylated, leading to the release of Bat3 and activates the downregulation of TCR signaling and suppression of T-cell proliferation and survival [126]. In ccRCC, TIM-3 and PD-1 co-expression on CD8 T cells is associated with worse outcomes including higher TNM stage, larger tumor size and lower PFS [127].

Lymphocyte activation gene-3 (LAG-3, also known as CD223) is a cell surface molecule that belongs to the immunoglobulin superfamily and is located near CD4. Like CD4, LAG-3 binds to major histocompatibility complex-II (MHC-II) on antigen presenting cells (APCs), but with a much stronger affinity [128], which prohibits the binding of the same MHC molecule to TCR and CD4, thus directly hampering TCR signaling in immune response [129]. LAG-3 is expressed in the membrane of multiple immune cells, including CD4 T cells, CD8 T cells, and T-reg cells. Several studies have delineated that LAG-3 is over-expressed on tumor-infiltrating CD8 T cells in various tumor types, including renal cell carcinomas [130]. LAG-3 overexpression leads to CD8 T cells exhaustion and resistance to anti PD-1 inhibitors [131]. This interaction occurs without binding to MHC-II, which have given rise to the discovery of additional tumor-related ligands, such as galectin-3 and liver sinusoidal endothelial cell lectin (LSECtin). These ligands seem to play an important role in the TME, although it remains unclear [132]. LAG-3 expression tends to be associated with a lower OS in RCC [120,133].

T cell immunoglobulin and ITIM domain (TIGIT) is a membrane protein with an extracellular IgV ligand-binding domain and an intracellular immune-receptor domain. TIGIT is primarily expressed on T cells and NK cells and binds to the poliovirus receptor PVR (CD155) and Nectin-2 (CD112) as a competitor to DNAM-1. DNAM-1 enhances cytotoxicity of T lymphocytes and NK cells, and TIGIT blocks its function acting like an immune suppressor. TIGIT has been found to be expressed on subsets of exhausted intratumoral CD8+ T cells [134,135].

## 4. Discussion

Resistance to systemic therapies in RCC, either intrinsic due to presence of resistance genes or acquired after initial tumor regression can directly impact the clinical course and additional treatment approach of these patients. This review highlights the new insights into key biological pathways underlying treatment resistance.

At the beginning of this century, treatment with TKIs that block the VEGFR has revolutionized the RCC treatment landscape, resulting in a significant increase in terms of life expectancy and quality of life for these patients. However, the benefit shown by these initial treatments was limited.

Looking at initial resistance to VEGFR2 inhibition by enhanced activity from other VEGFR receptors, multiple VEGFR inhibitors have been designed trying to overcome this obstacle. Moreover, the inhibition of the PI3K/Akt/mTOR pathway has become an option to overcome PTEN downregulation. Thus, preclinical studies combining sunitinib with PI3K/mTOR inhibitors, mTOR inhibitors or pan-AKT inhibitors, can restore sunitinib effect and induce apoptosis in those PTEN-negative cells [136,137]. However, in the clinical setting these combinations were related with increased toxicity requiring dose attenuation, and efficacy was less than expected in comparison with single-agent sunitinib at full doses [138].

In the FGF overexpression setting, lenvatinib, an oral inhibitor of FGFR, VEGF 1-3, PDGFR α, RET, and KIT, is able to overcome the FGF resistance mechanism and has demonstrated activity in the first line setting in combination with pembrolizumab and in subsequent treatment lines in combination with everolimus of patients with advanced RCC [139]. Inhibiting the FGF pathway with brivanib (a first-class dual inhibitor of VEGR2-3/FGFR1-2-3) in mice with pancreatic neuroendocrine tumors has resulted in promising activity after failure to anti-VEGF treatment [140].

Other TKIs have been developed in the last few years. Cabozantinib has been designed as a multi-tyrosine kinase inhibitor against VEGFR, KIT, RET, Tie2, cMET, and Axl inhibitor among others. Molecular testing from tumor samples by Zhou et al. demonstrated that cabozantinib could suppress Axl and MET activation including AKT and ERK downstream cascades induced by chronic sunitinib treatment [30]. Therefore, cabozantinib has been included in the therapeutic algorithm of patients with advanced RCC [141]. Crizotinib, a MET inhibitor, has been also studied in combination with axitinib, showing decrement in vascularity density along with suppressed tumor growth [142]. The role of crizotinib has been focused on the subtype papillary RCC due to its MET inhibition, but clinical results have not shown greater antitumor activity over other TKI VEGFR driven [143].

New pathways are being explored in order to reverse the resistance to TKIs. Ang/Tie pathway has indeed become an interesting target for new drug development, as MEDI 3671 (a monoclonal antibody against Ang2), trebananib (fusion protein which hampers the binding of Ang1/2 to Tie ½) or CovX bodies have demonstrated the ability to inhibit tumor growth and decrease vascular density [144,145,146,147].

Alternatively, the regulation of epigenetic alterations has also been spotted as a target. Tazemetostat is an EZH2 inhibitor studied in multiple solid tumors with promising results [148,149].

Lysosomal sequestration is a reversible resistance mechanism. A study conducted in sunitinib resistant RCC cells revealed that lysosomal function was suppressed when sunitinib was withdrawn from the cell cultures and drug sensitivity was retrieved [150]. Furthermore, alkalinizing lysosomes with an H+-ATPase inhibitor like bafilomycin has been also studied for reversing sunitinib resistance since pH gradient plays a key role in its sequestration. Notwithstanding, the excessive toxicity of this molecule constitutes a hindrance for its use in vivo. Following this rationale, chloroquine is being studied in preclinical assays showing interesting results in pancreatic neuroendocrine tumors (P-NET) combined with sunitinib [151].

Looking at the future, ncRNA expression and SNPs seem to be new paths to explore in further years. Long noncoding RNA lncASRS targeting with locked nucleic acids has provided evidence that could overcome the resistance and restore sunitinib response. However, further studies are needed to elucidate the role of lncASRS as potential therapeutic target as well as a clinical biomarker [57].

TME modulation has gained strength as a strategy to overcome resistance to TKIs. Pericytes have been conceived as interesting new targets to design novel drugs [66]. Pericyte coverage is regulated by PDGFs family molecules and inhibiting PDGFRβ in combination with antiangiogenic drugs can reduce pericyte coverage and inhibit tumor growth in mouse model P-NETs [152]. However, decrement of pericyte can likewise increase risk of metastatic dissemination and these strategies should always live in an intricate equilibrium where tumoral progression can be favored. Moreover, TME regulation focusing on the tumor endothelial cells with new molecules targeting the Ang-2 pathway and DII4 inhibitors have demonstrated anti-tumor activity in sunitinib and sorafenib resistant RCCs [62,63,153].

In recent years, strategies enhancing the immune system with the inhibition of immune checkpoint proteins PD-1/PDL-1 and CTLA-4 have revolutionized the RRC therapeutic landscape [128,140,154,155,156,157]. Nevertheless, there is still an important number of patients who never benefit from these treatments or lose this benefit in a short period of time. Taking this in consideration, big efforts have been taken in order to shed some light on the resistance mechanisms which lead to tumor insensitivity to ICIs and disease progression.

Novel immune checkpoints (such as TIM-3 and LAG-3) have been analyzed as potential targets, due to their responsibility in lymphocyte exhaustion and tumor immune evasion. Thus, TIM-3 has been targeted alone or in combination with anti-PD-1/PD-L1, with four ongoing phase I trials assessing antiTIM-3 antibodies in metastatic solid tumors (NCT02608268, NCT02817633, NCT03099109, and NCT03066648) [158].

Furthermore, several clinical trials targeting LAG-3 (alone or in combination with anti PD-1) in metastatic solid tumors including mccRCC patients are ongoing [159]. Relatlimab, an anti-LAG3 antibody with promising results in metastatic melanoma, is under investigation in combination with nivolumab (NCT02996110). Eftilagimod-α (IMP321), a soluble LAG-3 immunoglobulin fusion protein agonist has been evaluated in a phase I clinical trial, showing a promising activity inducing memory CD8+ T cells, as well as an acceptable toxicity [160]. XmAb22841, a bispecific antibody targeting CTLA-4 and LAG-3 is being evaluated in monotherapy or combination with pembrolizumab in select patients with advanced solid tumors, including mccRCC (NCT03849469).

Other immune checkpoints are under research. In phase Ia/Ib and randomized phase II clinical trials, tiragolumab (an anti-TIGIT antibody) had a tolerable safety profile with promising efficacy (most notably in patients with non-small-cell lung cancer), and clinical trials designed to assess the safety and efficacy of TIGIT inhibitors in patients with RCC are currently ongoing. An early-phase trial exploring a V-domain immunoglobulin suppressor of T cell activation (VISTA) inhibitor in patients with advanced-stage solid tumors is also ongoing [161].

Additionally, IDO-1 targeting has been one of the most promising approaches in the last years. The phase I/II ECHO-202/KEYNOTE 037 where the combination of the oral IDO-1 inhibitor epacadostat and PD-1 inhibitor pembrolizumab was tested, result in an objective response in 25 of 62 patients (40%), including eight complete responses and 13 patients with stable disease. In the mccRCC set, two patients presented responses out of 11 [162]. However, the ECHO-301/KEYNOTE-252 phase III study (epacadostat + pembrolizumab vs placebo in patients with unresectable or metastatic melanoma) failed to improve PFS or OS [163]. These results have led to the withdrawal in the development of IDO-1 inhibitors for the moment.

IFNγ pathway activation has been pointed out for its important role in sustaining the immune response. STING and RIG-1 are basic mediators in the detection of cytosolic DNA. The STING pathway activates nuclear factor-kappa B (NF-κB) and interferon regulatory factor 3 (IRF-3) through the activity of IκB, enhancing the IFNγ pathway and increasing the production of proinflammatory cytokines [164]. RIG-1 contributes to the stimulation of the immune system, favoring the production and activation of NK and CD8+ T cells [165]. Two phase I trials evaluating a STING agonist and a RIG-1 agonist as monotherapy or in combination with ICI respectively, in patients with metastatic solid tumors including mccRCC are ongoing (NCT03010176 and NCT03739138).

IL-2 is another promising target in the horizon of renal cancer treatment. Decades ago, high-dose IL2 was commonly used to treat mccRCC, achieving complete and durable responses in a subset of patients. However, the life-threatening toxicity associated with high-dose IL2 restricted this therapy to a limited number of young patients without underlying comorbidities. Bempegaldesleukin is a pegylated IL2 which preferentially binds to the beta-gamma subunit of the IL2 receptor. This interaction has shown a promotion of IL2 effects on T-effector cells, enhancing the expansion of effector elements, as well as depletes intratumoral T-reg cells. In phase I studies, bempegaldesleukin has been well tolerated with low grade 1-2 manageable adverse events, such as hypotension and edema. Despite clinical efficacy in randomized trials has still not been proven, data from tumor and blood analysis support the combinatorial use of bempegaldesleukin with ICI [166,167]. Other studies evaluating the utility of modified versions of IL2 and combinations with ICIs are also ongoing (NCT03861793, NCT03875079, NCT02989714, and NCT02964078).

Macrophage reprogramming is another promising approach nowadays, as diverse therapeutic strategies have been suggested to suppress tumor-associated macrophage recruitment, switching them back to the antitumor M1 phenotype [121]. Nevertheless, several studies have reported that high M2 macrophage tumor infiltration is associated with a more durable response to anti-PD-1 therapy [116,168]. This association was not found in patients treated with TKIs. Colony stimulating factor 1 receptor (CSF1R) expression has a key role allowing the switching of M1 macrophages into M2 tumor-associated macrophages [169]. Combinations of CSF1R inhibitors and ICI are under investigation in phase I trials (NCT02718911, NCT02526017).

Personalized neoantigen-based vaccines are a new compelling immunotherapy approach. Neoantigens are products of diverse tumoral mutations that can trigger tumor-specific T cell responses since they are exclusively expressed by cancer cells, thus avoiding vaccine “off target” effects. They can also propel immunological memory that boosts long term responses and delay disease recurrence. Despite being associated with a moderate tumor mutational burden, RCCs have an important proportion of frameshift indels and T cell infiltration, and are likely to have several candidate neoantigens for vaccine development. Phase I clinical trials with neoantigen-based vaccines in combination with ICIs or IL2 enhancers are currently being explored in RCC (NCT02950766, NCT03289962, NCT03548467, and NCT03633110) [170].

Finally, precision immunotherapy targeting surface antigens with chimeric antigen receptor (CAR) T cells and MHC antigens with tumor infiltrating lymphocytes (TILs) are under early development in RCC (NCT02830724, NCT03393936, and NCT03638206) [161].

Probably, combination strategies between novel immunotherapies and approaches in combination with “older” treatments such as TKIs could reverse the resistance mechanism in RCC. However, it is necessary to point out, that these investigational combinations with positive results in vitro/in vivo have to demonstrate efficacy and safety in further clinical trials. Moreover, we need to develop predictive biomarkers to current therapies in order to guide clinical decisions.

Predictive biomarkers of response to this target and immune-based therapies have been largely studied and have become one of the major challenges in ccRCC treatment. Currently, only the IMDC risk model (based on clinical features and initially designed as a prognostic model) has been validated as a robust tool for treatment selection not only for immunotherapy but also for TKI treatment [171,172,173,174,175,176,177,178,179]. Despite the PD-L1 expression and tumor mutational burden (TMB) have been broadly studied in many other tumors as a ICIs predictive biomarker, their applicability in ccRCC have not been demonstrated, mainly due to their unclear cutoff for positivity, intratumoral heterogeneity and inconsistency between primary tumor and metastasis [172]. Other promising predictive biomarkers have not bridged the investigational and clinical stages yet. Among these, neutrophil/lymphocyte ratio (NLR) [173,174], PBMR and molecular gene signatures [175,177] are worth highlighting.

## 5. Conclusions

New therapeutic options for RCC have expanded rapidly over the past decade, with the combination of TKIs and ICIs being the new cornerstone. Understanding the underlying resistance mechanisms to these treatments is a driving force for survival improvement in metastatic RCC.

Counteracting alternative modes of vascularization, EMT, lysosomal sequestration, and alternative molecular pathways can overcome TKIs resistance and restore sensitivity to these molecules. Tumor microenvironment modulation constitutes another fundamental approach, since it participates in both resistance to TKI and ICI. Finally, novel immune checkpoints like LAG-3 and TIM-3, as well as a renewed approach in cytokine therapy with IL-2 are promising targets in development.

Further investigation is warranted to improve our knowledge of RCC biological behavior and to develop successful treatment approaches.
